# Subjectivity as an Emergent Property of Information Processing by Neuronal Networks

**DOI:** 10.3389/fnins.2020.548071

**Published:** 2020-09-23

**Authors:** Riccardo Fesce

**Affiliations:** Department of Biomedical Sciences, Humanitas University, Pieve Emanuele, Italy

**Keywords:** consciousness, neuronal processing, emergent properties, neuronal network, subjectivity

## Abstract

Here, we examine subjectivity and consciousness as *emergent properties* of the computational complexity of information processing by the brain, rather than metaphysical phenomena. While Psychology concentrates on the emergent properties and Neurobiology examines the properties of the biological substrate, Neurophysiology and Cognitive Neuroscience link the two levels by investigating the mechanisms and processes by which the functions of the brain *emerge* from the anatomical, cellular and network properties of the nervous system. Our purpose here is not to locate the neural structures that sustain subjectivity or other psychic functions; rather, we examine the operating modes of neurons and neural circuits: they reveal an intrinsically relational quality; sensory elaboration itself proves to be relational and self-centred, necessarily associated with the vital, hedonic, emotional relevance of each experience and external cue, and intrinsically oriented to a behavioral interaction with the latter. The hippocampus adds to this self-centred relational perspective the capability of transforming the identification and the spatial location of objects into a contextualized representation of reality. Since the hippocampus is strongly interconnected with the archaic structures that evaluate vital and hedonic relevance and generate emotional responses, the contextualized information, emotionally colored, is transformed into a comprehensive individual experience. This way, a subjective, self-centred, affectively colored perspective arises in animals due to the intrinsic properties of neuronal circuits in the brain. We conclude that neuronal network processing is strongly characterized *per se* by a relational and self-centred (*subjective*) and emotionally colored, motivationally oriented (*personal*) perspective. The properties and features of neural processing discussed here constitute well-established knowledge in the neuroscientific community. Yet, from a layman’s perception, subjectivity still mysteriously arises in our brain due to the action of consciousness, and in epistemological and philosophical debates, the question often arises as to how consciousness may add the subjective and personal perspective to neural elaboration. The answer appears to be simple: it does not; subjectivity is already there, present *ab initio* in neuronal processing and not added *a posteriori* by some other “consciousness” function of unclear neural basis.

An impressive wealth of literature has been accumulating in recent years on the neuronal substrate of consciousness. The matter remains challenging, since “consciousness” is an umbrella term that subsumes a number of logical, representational and executive functions, which obviously are performed by many distinct circuits and computational modules in the brain.

In addition to the many computational aspects involved in consciousness, the term carries with it a metaphysical flavor, related to its intrinsically intentional, subjective and personal nature. In fact, from a layman’s perspective the essence of consciousness seems to lie in its capability to add the dimension of *identity* and *subjectivity* to the elaboration performed by neural circuits. If this were true, we might even achieve a clear and mechanistic knowledge of how the brain builds a precise, detailed and holistic, consistent internal representation of reality (the epistemological aspect of consciousness), but the question would still stand as to which circuits may generate a “conscious principle” that subjectively “observes” such internal representation (the metaphysical aspect of *reflexive consciousness*); one may conclude that no neural mechanisms might ever do this, and some non-neural (possibly metaphysical) principle must be involved.

In this paper, a different perspective is adopted: the modes of information processing by neuronal circuits are examined to explore to what extent a subjective and personal perspective might arise *as an emergent property* of the complex neural networks in the brain. This analysis reveals an intrinsic *relational* nature of neuronal elaboration; in particular, such relational nature appears to be inherently *self-centred*; furthermore, all activities in the brain (anything which is sensed/experienced) are analyzed and perceived in terms of their vital, emotional and operative relevance *for oneself*. This suggests that a *subjective* dimension (self-centred relational analysis) and a *personal* perspective (emotional, affective, operative relevance for the self) intrinsically characterize cerebral activity, and are not “added” *a posteriori* by some subjective observer function (consciousness) to an initially detached, objective representation of reality.

An effort has been made to clearly separate, in what follows, the data present in scientific literature from the views and hypotheses of the author.

## The Operating Mode of the Neuron

### The Layman’s Perspective

A simplified view of the operation of a neuron suggests that it receives a number of inputs (activation of the synapses on its dendritic tree and soma), which generate electrical events on its membrane; these electrical events possibly generate a nerve impulse (spike, action potential) if their sum produces a depolarisation that overcomes a certain threshold level (Bachatene and Bharmauria, 2017).

### The Data

Although this may be true, in principle, the situation for central neurons is much more complex and intriguing: the “language” of the neuron is not the impulse (i.e., its being *ON* or *OFF*), but rather a continuously changing *pattern* of impulse firing. Similarly, it receives a complex, continuously varying combination of momentary depolarising or hyperpolarising pulses at each of its synapses, that reflects the combined firing pattern in a large number of neurons in a cortical circuit (a “complex pattern of neural activity”): as a consequence, the response of the neuron does not monitor the overall intensity of synaptic activation, but rather the synchrony, consistency and continuous interaction among the activities of all the neurons that synapse on it. Cortical neurons examine the many inputs they receive with a degree of discrimination – precision, timing and selectivity – that depends on how narrow the time interval is for effective summation of the incoming signals (Wang et al., 2010); and such time window markedly changes, depending on the mode in which information reaches the cortex from the thalamus (Tremblay et al., 2016). The thalamus is in turn regulated by projections from the cortex (Mease et al., 2014), so the corticothalamic dialogue determines whether a circuit examines the incoming information in an approximate way (during inattentive processing) or in a more precise and discriminative manner (see below for a more detailed discussion; Guo et al., 2017).

### An Interpretive Hypothesis

Figuratively, it is as if the neuron listened to the melodies that are played simultaneously by many other neurons on its synapses. It would then elaborate, based on all this, an overall emerging melody (its own time-varying firing pattern); depending on the dialogue between each cortical circuit and the corresponding thalamic neurons, such melody would either reproduce the general tonality and rhythm of the incoming signals (in “non-discriminative” mode) or rather produce a more sophisticated elaboration of specific melodic and harmonic details in the concert it is listening to.

Such a way of integrating incoming signals clearly indicates that no central neuron ever responds to a datum (be it of sensory or of endogenous origin), but they detect specific *relations* among the *patterns* of activity of the neurons that are connected to them, and may thus attribute a consistent (vague and generic or more specific) meaning to such complex patterns of activity.

### The Data

The property of detecting patterns in the relations among sensory data is particularly well documented by recordings performed in central neurons positioned along the cortical paths that analyze visual information.

**FIGURE 1:**
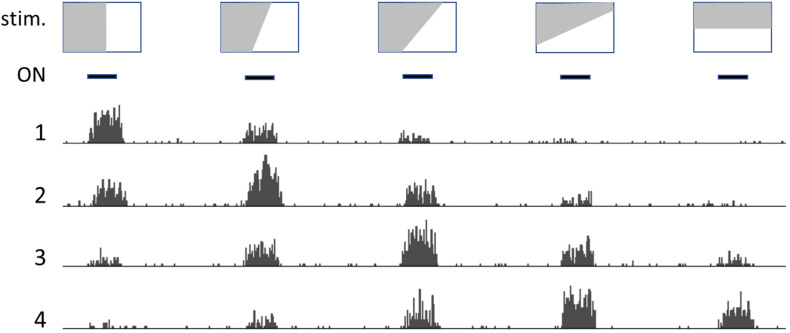
Drawing that illustrates how *neurons detect relations* (patterns). Depiction of the activity of neighboring neurons in the visual cortex: top row shows representative images, that are presented one after the other at the times indicated by the black bars (ON, second row). Numbers 1 to 4 represent histograms of spike frequencies in recordings from neighboring neurons. Each neuron preferentially responds to a profile with a specific slant angle. However, pattern detection is not crisp and absolute, but rather fuzzy and “tentative.”

Neighboring neurons in occipital-temporal areas respond to patterns in the visual field, such as lines or borders with variable slant (Figure 1); each neuron generates a firing rate that depends on how closely the slant of the line/border approaches the angle it is designed to recognize, with neighboring neurons responding to slightly different angles in an ordered way (original observations by Hubel and Wiesel, recollected by Alonso, 2009).

This occurs somewhat independently of the precise location of the line in the visual field, which indicates that these neurons signal the presence of a specific pattern in the incoming visual information, rather than precisely locating cues in the visual field. In more anterior areas of the temporal lobe, neurons display increasingly sophisticated elaboration of the incoming image, and will fire in the presence of any visual pattern that may represent a relevant object, such as a hand or a face (Gross, 2008).

**FIGURE 2:**
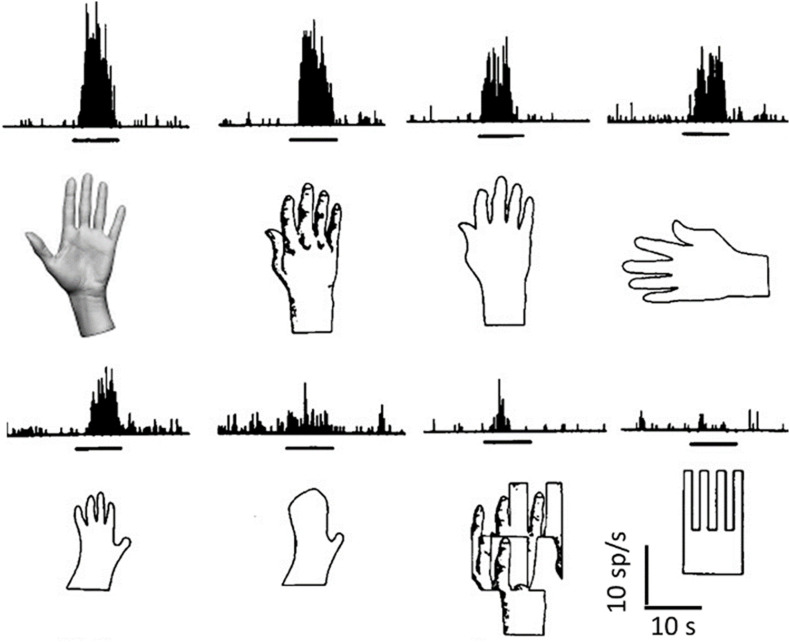
Object recognition by a single neuron. Responses (histograms of spike frequencies vs. time) of a single neuron to the corresponding images, presented during the times indicated by the horizontal black bars. Many different elaborations clearly converge onto this neuron, which does not recognize a pattern, but many possible visual patterns that can be related to the shape of a hand, through association and generalization, either in a pictorial or in a structural way (elements and their relations). Object detection is tentative, and the discharge rate indicates the likelihood that what is being seen belongs to a certain conceptual category. Modified from Gross et al. (1972).

Although neurons innately programmed to recognize specific relevant shapes are present in the temporal lobe (Figure 2), for most of the objects that we recognize, this cannot occur by means of innately wired connections.

### A General Interpretation

Returning to the above musical metaphor, if we were able to encode a complex pattern of neuronal activity into audible music, then that music would indicate that something has been recognized, i.e., the “awareness” of something has arisen. Zeki (2001) coined the term “micro-consciousnesses” to refer to these rudimentary elements of awareness, observed in visual elaboration circuits. Note that if this same metaphor is applied to the auditory system, the “music” played by a neuron would not reproduce the heard sound; it would rather signal a specific feature in the auditory input stream (a note, a chord, a phoneme or a specific sequence of phonemes).

The occipital-temporal path of visual elaboration is called the “what pathway,” but elaborations of auditory, somatic and sensory-motor information also converge with this path to contribute in recognizing “objects,” and even in associating (and memorizing) the sound of words (and the shape of written words) to objects.

The recognition of objects arises from the capability of each neuron to generate a specific response when the complex activity patterns of many other neurons points to the possible presence of an object. This process of object recognition may be either precise and reliable, when the neurons are in a discriminative processing mode, or quite tentative and uncertain. In either case, an object is recognized if a neuron (or a set of neurons) specifically reacts to the pattern of neural activity elicited by the presence of the object, as if it were designed to recognize it.

## Emotions and Learning

### The Data

At every moment in our life, a number of deep subcortical structures (hypothalamus, amygdala, midbrain ventral tegmental area, raphe, nucleus accumbens, substantia nigra; see e.g., Bayer and Glimcher, 2002; Balleine and Killcross, 2006) monitor the activity in most areas of the brain and evaluate of the relevance of the current situation for survival and well-being, i.e., the vital, emotional, hedonic and operational relevance of external cues and/or the current experience (e.g., Robinson et al., 2016). The activation of these structures produces visceral-vegetative responses (change in heartbeat, sweating, weeping, shaking…) and changes in posture and facial expression (smiling, looking sad, frightened, angry…), but it also activates the limbic areas of the cortex that enable the emotions (fear, pleasure, grief, rage, hope, disgust…) to be felt and cognitively elaborated by the other appropriate areas of the cerebral cortex (Damasio, 1996).

Emotional relevance influences the network: any novel pattern of neuronal activity tends to induce changes in synaptic connectivity and strength, so that it is likely to become more easily reproduced; such *plastic* changes typically occur when a pattern occurs repetitively, or it is associated to a concurrent emotion (detection of vital relevance). The activity at synapses, and in particular the precise association and reciprocal temporal relations among the signals that converge on a neuron, produce modulation of numerous biochemical processes, at the presynaptic nerve terminals as well as at the postsynaptic specializations; this introduces temporary and reversible changes, but also permanent and irreversible ones, in the structure and efficacy of synaptic contacts and therefore in the overall connectivity of the local network (“synaptic plasticity;” Feldman, 2012).

### An Interpretive Hypothesis

So, how does the brain become able to “recognize” objects the circuits are not initially wired to recognize? The amount of activity converging on a neuron is not as important as the precise way the combination of time-varying synaptic activations interact at each moment on each neuron; in fact, depending on the precise timing of converging synaptic inputs, distinct molecular events may occur and trigger biochemical modifications, in addition to the electrical events. The resulting modifications in the efficacy of the synaptic contacts change the way the neuron processes the incoming information (long term potentiation or depression). These “plastic” changes occur as a consequence of specific sequences and time delays between the activations of neighboring synapses.

Through neuronal plasticity, any novel pattern of neuronal activity, i.e., any unknown element of the current experience, can be associated to existing knowledge: it will receive a cognitive meaning (logical, causal, operational) based on its relations with known elements in the experience (similarity in neuronal activity patterns); it will be attributed a *personal* meaning (connection with some vital value) related to the emotions associated with such known elements, with the current experience and with its cognitive/operative implications (opportunities, risks, possible developments). This way the circuitry gets to “know” – and becomes able to detect – the novel elements in the experience, to attribute a meaning to them and to re-cognise them in the future. Once more, this process is totally based on the capability of neurons to examine relationships, consistencies and reciprocal interactions.

The specific features of neuronal plasticity accounts for two main quite relevant consequences: first, in general, the neural circuits change the way they process information as a consequence of the information itself they elaborate; second, the plastic modifications have intrinsic *associative* characteristics, in that they occur depending on the precise spatial and temporal relations (association) among synaptic activations on the neuron. All this accounts not only for all forms of associative learning (classical, aversive and operant conditioning, procedural learning, associative memory), but also for the fact that each brain elaborates the same information in a different, specific way that arises from all the information it has processed up to this moment and – more relevant – the *way* it has elaborated such information. This is a strong basis for the *emergence* of *individuality* as a consequence of the mode itself of operating of neural circuits.

Depression and pruning of synaptic contacts is at least as relevant as long-term potentiation: the number of synapses in the brain markedly decreases in development, and especially so during adolescence. This trims the associative network: cerebral elaboration matures from the childish generic and mythological perspective toward the more precise, rigorous and rational adult attitude.

This perspective on neural plasticity lets us take a novel perspective not only on individuality but also on neuropsychological disorders.

**FIGURE 3:**
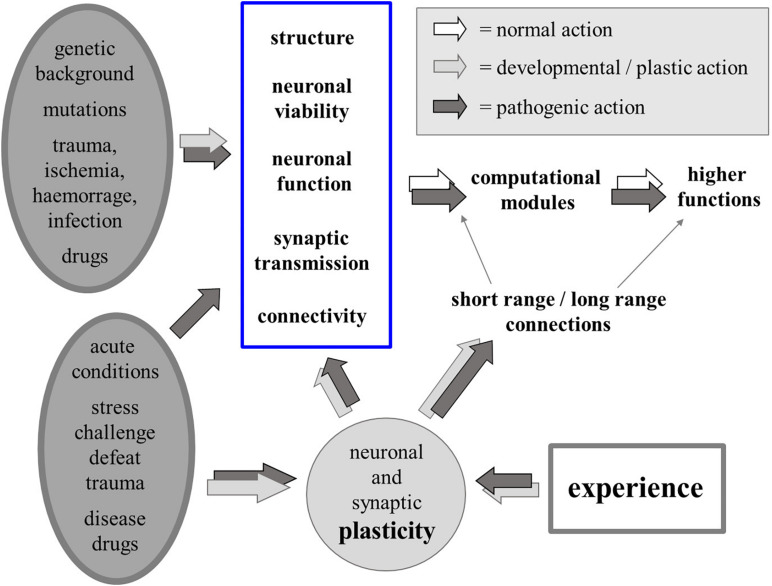
Physiological and pathological biological factors (chronic or acute; left-hand side) may alter neuronal structure, viability, function and connectivity, affecting the function of computational modules in the brain and short as well as long-range connectivity. Due to experience-driven neural plasticity, “biological” (organic) and “psychogenic” (experience) factors may similarly affect the function of the computational modules and the higher functions that result from the interactions among such modules.

We know that a number of biological factors – genetic, developmental, pathological or iatrogenic, acute or chronic – may act directly on neuronal structure, viability, function and connectivity, affecting the function of computational modules in the brain and of the higher functions that result from the interactions among such modules. Since experience also influences all aspects of neuronal plasticity, most changes that can be produced by biological causes can also be produced by neuronal activity itself, i.e., by experience, thus blurring the border between the organic/biological “Nature” of the self and the psychological “Nurture” that molds identity and possible psychic disorders (Figure 3).

## The Self-Centred Relational Perspective

### The Data

In addition to being processed along the “what” pathway, all sensory data are also examined in terms of spatial relations, both reciprocal ones and with respect to the sense organs that captured them. This is a particularly challenging process, because the location of a stimulus perceived by the eyes will be mapped as a function of gaze direction, an auditory stimulus will be located with respect to the head, and a somatosensory stimulus with respect to the position of the limb or extremity that senses it. The resulting, modality-specific, sensory spatial maps are organized in the superior colliculus (a midbrain structure) and relayed to the parietal cortex, which puts in register these maps with each other and remaps the stimulus – whatever its sensory origin was – in terms of movement, i.e., the direction that the gaze, the head, a hand or other appropriate parts of the body are to be directed to in order to adequately face the stimulus. Such further mapping is bounced back to the superior colliculus, which controls rapid and reflex eye and head movements (Gandhi and Katnani, 2011). Notice that this reflex is extremely rapid and is brought about by subcortical structures (colliculus and tectospinal motor system): the cortex would be much slower in reacting.

**FIGURE 4:**
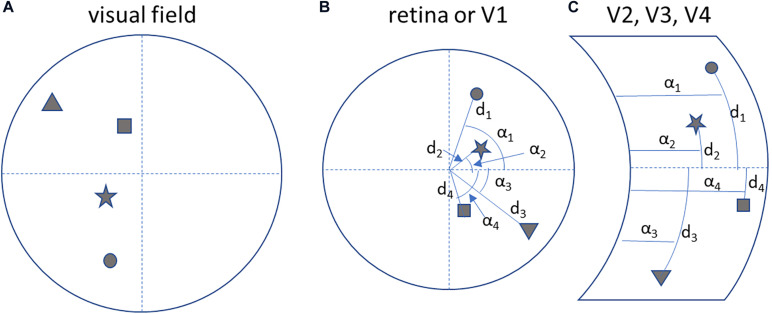
Neuronal processing is *self-centred* and *interaction oriented*. **(A)** A hypothetical visual field. **(B)** The corresponding topological mapping of the image on the retina and on the primary visual cortex (V1). **(C)** The transformation of the topological mapping in the right associative visual cortices (V1 to V4): notice the transformation to a spherical coordinate system (angle α_n_ and distance d_n_), that helps to map objects in terms of the direction (azimuth and elevation) of a movement to reach them.

Note that in the associative visual cortices in the occipital lobe the visual field is mapped after a transformation from Cartesian to spherical coordinates (see below, Figure 4; Tootell et al., 1997).

Simultaneously, higher processing of the spatial relations occurs in the parietal cortex, along the occipital-parietal path (the so-called “where” pathway). Here, spatial relations are analyzed. The geometric and mathematical analysis of the physical space, as well as any virtual spaces, is performed by these circuits, which actually contribute to elaborate any complex sets of sensory or mental data and generate the idea itself of space, as a set of reciprocal relationships among elements that are simultaneously present.

The capability of the parietal cortex to remap all sensory inputs in the surrounding space, according to a motor map, enables these circuits to directly activate premotor areas in charge of programming movements (Maranesi et al., 2014). In particular, one set of neurons (so-called canonical neurons) are activated to prefigure the movements (reaching, grasping, rejecting) needed for the appropriate behavioral interaction with the identified object (“affordance”); here, “appropriate” refers to those interactions that have been positively reinforced during past experiences, since they were successful in producing pleasure, reducing pain or nuisances, or helping to operationally achieve an aim. Another set of premotor neurons (so-called mirror neurons) are also activated, to prefigure the appropriate *finalized action* (motor act with a purpose) that can be performed with the object (holding it, bringing it to the mouth, throwing it away…) (Fadiga et al., 2000); again, appropriate here means heuristically consolidated by reinforcement.

### A Theory on How to Interpret These Data

The mapping of the visual field in the associative visual cortices according to a spherical coordinate system (Figure 4) constitutes motor-oriented vectorial information about objects: direction and extent of a movement to reach for them. So the parietal cortex, in mapping objects in space, actually predicts possible motor behaviors to reach for them.

The elaboration of spatial relations by the parietal cortex is possibly the most powerful computational instrument in the cognitive representation and analysis of reality; in fact, humans tend to represent any complex problems by mapping them in some sort of virtual space: time is handled by visualizing it as a time line (Oliveri et al., 2009), numbers are visualized as a series in space, even emotions are given a spatial representation (a dear friend is internally represented as “close” and occupying a “larger room” in one’s visual field and inner life).

In the context of what is being discussed here, it appears that brain circuits examine and interpret every cue not only in terms of their internal and reciprocal relations, but also in terms of their relation *with one’s own body*, and of possible behavioral *interactions* with the cue: every cue is therefore given a meaning not only in terms of its possible vital relevance, but also with respect to its relevance to one’s own actions, aims and operative plans.

This way the interpretation of each cue becomes a clearly subjective one, colored by emotional valence, characterized by possible usefulness and appropriateness to achieve specific aims: it becomes a clearly *subjective and personal* interpretation.

### A Theory on “Autopilot” Behavioral Control

The parietal cortex is able to elicit the activation of motor programs in the premotor cortex in response to the detection of any possibly relevant object in the environment. Still, one obviously does not react to every cue they detect. The selection among possible cue-driven (“reactive”) behaviors is one of the main functions of the basal ganglia, deep brain structures that monitor cortical activities and return either facilitatory or inhibitory feedback onto each such activity, based on the hedonic value (pleasure) that presumably is associated to it, according to past experience (positive or negative reinforcement that such activity has encountered) (see, e.g., Averbeck and Costa, 2017). As long as nothing unexpected happens, this regulation of behavior, which might be defined as *cue-driven reactive behavior*, accounts for most of the activity of an animal, and does not actually need to be monitored by attentive and intentional control (awareness may be present of the actions that are performed, but it is not needed). A similar mode of behavioral control operates in humans as well, when they act with no attentive and intentional supervision, in a so-to-say *autopilot* mode.

Any unexpected event, error, failure, contradiction, inconsistency, puzzling aspect or unpredicted result generates an arousal response and activates the anterior cingulate gyrus, which generates a (possibly pleasurable or unpleasant) reaction of surprise, shifts selective attention to the problem and involves the prefrontal cortex in rationally facing the problem, by driving the working memory system to develop alternative, rationally elaborated, behavioral strategies (Carter et al., 1998).

Typically, “autopilot” behavior is guided by external cues rather than endogenous projects, and being regulated by the basal ganglia it occurs with no attentive and intentional control. Still, it is equally effectively guided by a personal, utilitarian perspective, by the nigro-striatal control of the basal nuclei. This does not imply that these behaviors are involuntary or “unconscious” (we may well be aware of what we are doing); it simply indicates that consciousness – attention and intentionality – is not needed to produce a behavior that is clearly directed in one’s own best interest.

## Transforming Information Into a Personal Experience

### The Data

The information about the meaningful objects detected in the environment (“what” pathway) and about the relations among objects and with oneself and the overall environment (“where” pathway) are relayed, respectively, to the perirhinal and parahippocampal cortices, and from these areas they converge onto the entorhinal cortex, the main gateway to and from the hippocampus. The hippocampus performs an overall integration of these two kinds of information by generating a spatial and temporal *context* (Eichenbaum, 2017).

The hippocampus is an ancient cortical structure that is much more developed than the neocortex in lower mammals (Murray et al., 2018); due to its above-described contextualizing function, it has a central role in sustaining spatial memory (Moser et al., 2008): in laboratory animals, its proper working can be tested by examining the performance in spatial tasks, such as learning to escape from a labyrinth or recognizing spatial clues. In humans, the temporal context is at least as important, in that it helps to interpret the how and why of an episode; similarly, the contextualization in virtual spaces – cognitive contextualization – makes it possible to link abstract notions (facts) into a context, which greatly helps in memorizing them. Notice that this process relies heavily on the capability of retrieving previously acquired knowledge as a framework for contextualisation. Thus, in humans the hippocampus has a crucial role in declarative (both episodic and semantic) memory (Opitz, 2014).

### A Theory on the Emergence of a Personal Experience

The hippocampus transforms a huge set of mutual spatial relations among objects into a contextualized perception of the environment (situation), and the temporal relations among events into connected and possibly causally related sequences (processes). This way an integrated, consistent, logically organized and dynamic internal image is generated of the external reality and of the episodes that arise from events and actions. Because of the great plastic properties of hippocampal circuits, such internal images can be saved and possibly recalled in the future to help analyze and interpret novel situations.

The hippocampus is strongly and bidirectionally connected to the amygdala, the main subcortical structure in charge of attributing a vital relevance to the current experience. This way, the memorizing capability of the hippocampus is enhanced whenever the current experience is recognized as emotionally relevant by the amygdala; meanwhile, the hippocampus helps in correctly evaluating possibly relevant clues by contextualizing them (a lion in a cage must not elicit fear).

More relevant to the topic at hand, thanks to the input from emotion-related subcortical structures, the internal picture of reality and of the current experience, generated within the hippocampus, becomes emotionally colored.

**FIGURE 5:**
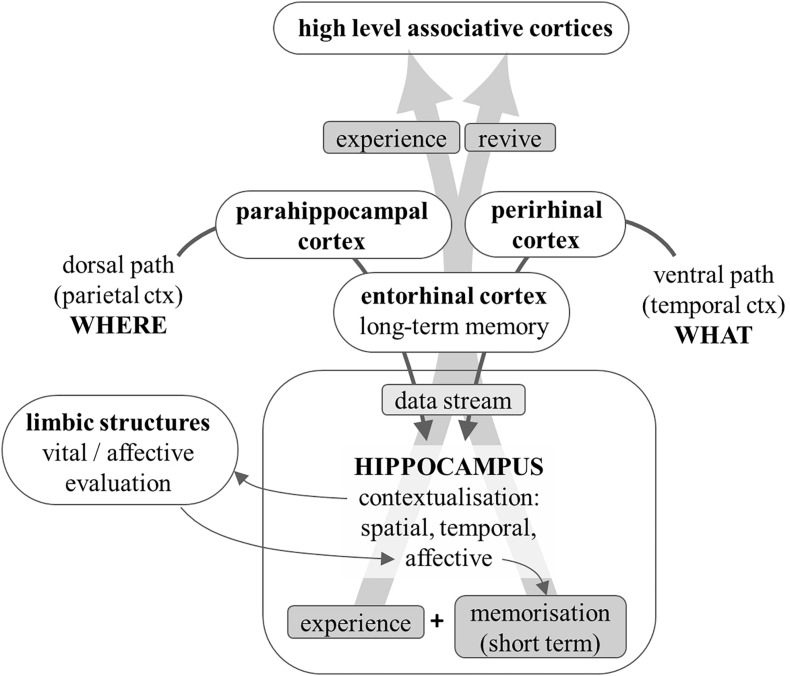
Diagram that shows how information is transformed into a *personal experience*. Data about object identification and location is relayed to the hippocampus. The latter provides contextualisation: spatial, temporal (which transforms sequences of events into episodes), and semantic. The strong bidirectional connections with limbic structures help the latter to emotionally evaluate the contextualized situation; the emotional coloring adds affective contextualisation and enhances memorization by the hippocampus. The experience so *personalized* is reflected onto the peri-hippocampal cortices that relay it to higher level associative areas and can store it as a long-term memory of the comprehensive and personal vital experience.

It can therefore be concluded that the hippocampus transforms the streams of data about objects, their emotional relevance and their reciprocal spatial relations, into a comprehensive, integrated, emotionally tinted, vital experience, i.e., a properly *subjective* and *personal* perspective of the current moment of one’s life. This integrated *personal experience* of the current moment, rather than the raw sensory data it was built upon, is relayed back by the hippocampus to the cortical areas involved in higher mental functions: we cognitively elaborate, and we remember, our personal experience of reality and of the episodes of our life, rather than what was effectively there and occurring, or what was *objectively* perceived by our senses (Figure 5). In simple words, this integrated personal experience simultaneously constitutes a *state of mind* and an *internal image* of an object, a situation, an episode, a fact/concept.

## Our Hypothesis on Emergence

The mode of neuronal functioning, the process of spatial and temporal summation of synaptic signals, the connectivity and the plastic properties of neuronal circuits give rise to a number of emerging “meta-biological” properties:

### From Information Processing to the Emergence of Knowledge

The firing pattern of a neuron reflects a real-time elaboration of the synchronicity and consistency of the time-varying inputs onto its dendritic and somatic synapses. Each of such inputs reflects the firing pattern of a neuron and represents the detection of a feature in sensory input (*external cue*) or endogenous cerebral activity (*internal cue*). Thus, the firing pattern of each neuron signals that (and to what extent) a set of specific features are present in the incoming information. Most neurons project to thousands of other neurons, so that each bit of information gets examined in many ways, in parallel, by many neurons that put it in relation with multiple sets of other data: as a result, information is continuously rearranged and read according to many interpretive criteria and attributed a meaning (perceptual, emotional, functional, operative) thanks to such logical reorganization, through associations and generalizations. This way information is transformed into meaningful information, i.e., into *knowledge*.

### Liking and Disliking, Desiring, and Fearing: The Emergence of a Personal Meaning

Every brain activity is bounced onto several deep structures: the amygdala detects vital and physiological relevance, activates visceral-somatic responses through the hypothalamus (the bodily component of emotions) and produces cortical arousal and possibly excitation or anxiety; the serotonergic median raphe nuclei control anxiety and mood, regulate the weight of social constraints, ethical merit and reality judgment in choices (cognitive as well as behavioral) and contribute to define the emotional response; dopaminergic structures in the midbrain perform hedonic and operative evaluation that guide spontaneous (*autopilot*) behavior through the basal ganglia and generate motivational drives for the prefrontal executive cortex. All these elaborations are fed to the limbic cortex to generate feelings, expectations and desires: each sensory experience, each motor and cognitive program and/or behavior are this way framed in an emotional and affective setting oriented to personal well-being and achievement of operational success and/or pleasurable outcomes. It should be apparent how this transformation can turn every pattern of neuronal activity into a personally meaningful event, and orient spontaneous as well as rationally programmed behavior toward the best interest of the self.

### The Emergence of Conceptual Elaboration

In order to abstractly manipulate a concept in an efficient way, a system of symbolic references (some form of language) must be there; however, the concept is formed in the course of information processing by neuronal networks before such symbolic system is in place. The existence of neurons in the occipital cortex that fire in response to the presentation of a line, or a circle, in the visual field indicates that each object that is perceived will be able to generate a specific pattern of activity in the brain, a specific “signature” in neural activity. Although this cannot be looked at as the signature of a “concept,” it might be thought of as a generalized “internal representation” of the object. The evaluation of possible vital relevance, the association of an emotional response, the activation of possible behavioral interaction programs, and the prediction of possible operative value for the identified object actually give it a “meaning.” Such meaning is distinct from the specific sensory properties of the object (its appearance, shape, size, structure, color, texture); rather, it is related to the concerted activity of a number of neural networks in the brain (neural signature), aimed at identifying the possible interactions with such an object, i.e., its subjective and personal relevance (meaning).

The association of a loud sound with the fear reaction it generates elicits a signature that is not specific to the particular sound, but will be shared by any other sufficiently strong, sudden, and therefore fearful, sound, and constitute the “internal representations” of a “fearful sound.” Such internal representations are general, abstract references to categories of objects/experiences, giving rise to “proto-concepts,” such as that of a “fearful sound,” even *before* any words are coined to refer to them. The same holds for a “hurting/burning/soothing object,” a “reassuring/menacing face,” a “pleasant/bitter/sour food.”

In terms of information processing, each of such proto-concepts consists in a specific pattern of concerted neuronal activity in various areas of the brain (signature). Each item referred to by the proto-concept will activate specific patterns of activity related to its particular sensory features, but it will also elicit the signature of the proto-concept, which therefore constitutes a mental representation, and *internal image*, of a *category* of objects, situations, experiences that share the same (or a similar) meaning for the self.

The “proto-concepts” here discussed have a clear pre-verbal nature; still, they can sustain a rudimentary form of pre-verbal thought, of non-verbal communication (by mimicking reactions/emotions) and even verbal communication (onomatopoeic, “boom” for a thunder, “meow” for a cat).

### The Emergence of Imagination

The hippocampus was mentioned to be able to generate “internal images” as consistent integrated pictures of what is being experienced, and save them in memory to possibly revive them, together with the emotional experience and the possible operative relevance associated to them. “Reviving” the experience simply means to be able to reproduce in the brain the neuronal activity patterns it generated in the first place. The reproduced patterns will obviously differ in that the primary sensory input that the actual experience originally elicited will not be there; this incompleteness can be detected by the nervous system and a corresponding modulation of (mostly serotonergic) projections from subcortical systems to the cortex will allow the brain to label them as “non-real,” *imaginary*. It is well known how serotonergic drugs – such as LSD – can impair this capability of the brain, giving rise to delusions and hallucinations.

The process of proto-conceptualization described above indicates that mental representations tend to depart from the mere reproduction of sensory experiences and rather be categorical, generic, non-specific. This is particularly important in attributing a meaning to novel sensory experiences: every aspect of the pattern of neuronal activity elicited by the novel experience will share some features with some patterns that have been experienced in the past and attributed some meaning (personal relevance); in particular, they will “resonate” with the signatures of some proto-concepts, and this will make it possible to interpret the novel experience and give it a meaning. Notice the quite relevant *active* component that this implies in the process of perception: sensory information will revive a number of activity patterns, associated to proto-concepts, and the attribution of meaning will be based on how the novel pattern “resonates” with the many imaginative productions awakened by the experience (a neural correlate of the “qualia”). The genericity, non-specificity of proto-concepts is a central feature of imagination, each mental representation possibly referring to many real targets (semantically pleiotropic, symbolic: notice the etymology of symbol, from συν+βαλλo, “throw together”): internal images are symbolic, imprecise, vague, incomplete and ambiguous; they may quite fluidly merge into one-another, unless rational thought, helped by a rigorous symbolic system such as language, intervenes in impoverishing their possible references and polishing them by reducing them to words with a (subjectively) specific and (hopefully) unambiguous meaning.

### The Emergence of Selective Attention

#### The Data

Information processing by cortical circuits can proceed in different modes. A synaptic signal (a depolarization) typically persists for a few milliseconds; in principle, a pyramidal neuron (the principal, projecting neuron in a cortical column) can accumulate membrane potential changes generated by all its synapses over a period of several msec; this implies that it will not be able to specifically recognize a precise time coincidence of the inputs it receives (and precise synchrony in the time-varying activation of its synapses) and generate in turn a firing pattern that precisely represents the structure of incoming data. However, most inputs coming from the thalamus (the mandatory last station for any information directed to the cortex) also hit the PV (parvalbumin positive) inhibitory interneurons that project on the principal neurons, so that any depolarization will be very rapidly (∼1 msec) followed by an inhibitory hyperpolarization. In this situation, synaptic inputs on the principal neuron can only summate if they occur within a very narrow time window, making the elaboration by the neuron particularly precise and discriminative (Wang et al., 2010).

Thalamic neurons display a complex behavior. When they receive no tonic depolarizing input, they respond to synaptic inputs by repetitively firing (“bursting”): this occurs during slow-wave sleep, but also during wakefulness, in areas of the thalamus that project to cortical circuits that are not targeted by selective attention. Thalamic bursting produces desensitization of PV interneurons: the principal neurons are no more immediately shut off, the time window for synaptic summation widens and they shift to non-discriminative elaboration mode. The bursting behavior of thalamic neurons can be switched off in a diffuse way by a number of subcortical circuits implied in the regulation of the wake-sleep cycle and general arousal; however, when a cerebral area is activated (targeted by selective attention), it is able to maintain the corresponding thalamic circuits in non-bursting mode, and this way can operate in a fully discriminative way (Mease et al., 2014).

For example, when a person is concentrated in reading visual cortical areas operate in a discriminative fashion, but the auditory cortex does not; if somebody talks to them, the auditory cortex becomes activated, shifts to discriminative mode, the focus of attention moves to the auditory input and the person, who has perceived the beginning of the sentence as a mere sound/noise, will now be able to understand the rest.

### The Emergence of Intentionality

#### A Historical Perspective

Selective attention is linked in many ways to consciousness. The phenomenological school (Brentano, Husserl) strongly emphasized the directedness (“aboutness”) of consciousness as an “*intentional*” (in-tend, tend toward) act, which needs to be directed to an object (the *content* of consciousness). For some Authors, intentionality is somewhat equivalent to a “mental representation,” which would make the common perception of consciousness a mongrel concept (Block, 1995, 2002) that involves *phenomenal* perception (*what it is like to*), possible *access* to selective attention (intentionality) and maybe a reflexive component; the latter, however, would require a brain capable of a certain degree of abstraction and (verbal) concept manipulation.

#### A Modern Educated Perspective

From a neurophysiological perspective, an experience corresponds to a certain pattern of activation of neural circuits in the various areas of the brain (signature). As mentioned above, the hippocampus is capable of reviving significant signatures experienced in the past, thus reproducing a virtual re-experiencing. Projections from subcortical structures help in discriminating whether an experience is real (here and now) or is a virtual re-experiencing in the absence of the corresponding sensory inputs (imagination).

Computationally, *phenomenal* consciousness can be looked at as the imaginative activity generated by experience (or endogenously produced). Access to this imaginative content would be granted by shifting the involved cortical areas to a discriminative mode of operation, i.e., focussing selective attention mechanisms on them. This would well account for *intentionality*, but not for the *reflexive* aspect of consciousness; still, the capability of distinguishing whether the object of a mental act is real (out there) or not (imaginative) constitutes an important step toward a judgmental (if not reflexive) perspective. Husserl suggested that *some* kind of reflexivity is essentially built into every conscious act, because the (intentional) object of a “mental act” (thought, judgment, desire) is perceived as external to the mind, out there or in some ideal realm, and therefore “transcends” the mind and its acts.

### The Emergence of “Here and Now”

#### The Puzzling Data

Back in the 1980 Libet et al. (1983) suggested, based on direct stimulation of the brain during neurosurgical operations, that it takes around half a second for a stimulus to work its way through to consciousness. Quite a lot of rumor was generated, some 30 years later, by the demonstration by Haynes’ group that signals in brain activity predict a person’s upcoming “free” choice up to several seconds before they believe they have made up their mind (Soon et al., 2014).

#### A Theory on the Awareness of Here and Now

Apart from philosophical considerations and the number of criticisms these data elicited, they raise a puzzling computational aspect: how can our brain drive us to swing a bat and correctly hit a baseball that may travel at up to 100 mph?

Let’s neglect the possible terrible delay of consciousness; simply due to the time needed for neuronal processing (at least 100 msec), our brain will localize the ball more than 4 meters behind its true current position; furthermore, the premotor cortex has to set up and launch the motor program to drive the bat some tens (possibly hundreds) of milliseconds in advance of the actual movement. This means that the circuitry in the brain must be able to predict the current position of the ball, based on an extrapolation of its movement (perceived with a delay), and program the movement in advance, so that at the moment of the impact (this precise instant, *now*) the ball and the bat come to meet each other at a position that the ball does not seem to have reached yet and the bat should have long passed (based on when we “commanded” it). This is not a particularly hard computational task, but it implies that reality has to be depicted by the brain by: (1) extrapolating past sensory inputs (events), to some “*now*” time; (2) considering motor commands issued some while back as if they were being issued exactly *now*; and (3) generating a *virtual* “*now*” *instant* in which the brain seems to perceive, as precisely synchronous, something it extrapolates from past sensations, what it has commanded way back and proprioceptive sensations, that inform on the state of muscles and joints, that have not arisen yet. Consciousness or not, the circuits are able to generate such *virtual now*, which manages to be perfectly in phase with what is actually occurring out there; and when this depiction of the changing reality by cerebral circuits is fed to conscious awareness (Herzog et al., 2016) the picture is not delayed: it simply reflects a reality that has been computationally recreated in brain circuits using prediction, extrapolation and appropriate delays, and only hopefully reflects what is going on out there *now*.

It is apparent that this process occurs in any brain that is capable of producing appropriately timed reactions to external stimuli (in every animal with a brain), so that the perception of “here and now,” of being there and interacting with reality, must be *computationally* present in any such brain as a “mental state” that produces an “internal image” of this precise moment. Telling a story about it, i.e., a proper explicit *reflexive awareness*, is another story, will require a more refined brain and a delay, needed for elaborating such mental state, but will be perceived (through the same trick of predicting and delaying) as if it were occurring in the same moment.

### The Emergence of Agency – Perception of One’s Own Body, Sensing, Acting, Thinking

As we become proficient in performing an activity, such as playing the piano, there remains no actual time to realize whether we are performing accurately or not. If we try to do so, the performance gets jammed.

#### The Data

The cerebellar circuits are much faster than the cerebral cortex, and very precisely and finely tuned in terms of timing; they are called into play whenever an inconsistency is encountered between programmed and actual movement, and they rapidly adjust intensity and timing of the activation of each muscle involved, so that the imprecision of the movement is not perceived from the outside or even internally. In doing so, cerebellar circuits undergo plastic changes (“learn”) so that repeated movements become “automatic,” perfectly organized and timed. In the meantime, cerebellar circuits “reassure” the cerebral cortex that everything is alright (Therrien and Bastian, 2019), tuning down the comparison of proprioceptive sensation with the motor commands issued (a rapidly corrected imprecision is not perceived). This way the cerebellum speeds up not only motor performances but also cognitive ones, such as repeating poems learnt by heart, ordering words in a sentence, picking the right tense of verbs or the appropriate pronoun to indicate a person or a thing, etc. (although the details of this function remain mostly inferential; Koziol et al., 2014). This role of the cerebellum is essential in order to preserve the capability of cerebral circuits to generate the consistent perception of *now* in the face of an accelerated performance.

The consistent perception of *now* is essential for consciousness unity: specific disorders of the cerebellum have been reported to be associated with dissociative conditions, i.e., disorders of consciousness such as autism and schizophrenia (e.g., Turner and Schiavetto, 2004). Experimentally, consciousness can be deranged by procedures that produce some forms of “disembodiement”: in the “rubber hand illusion” situation, subjects experience an artificial hand as part of their own body, while the real hand is subject to a sort of disembodiment (della Gatta et al., 2016); similarly, when subjects were shown a view from a video-camera positioned two meters behind their back through a head-mounted device, drift in perceived self-location, self-identification with the virtual body, and touch referral to the virtual body were observed (Cowie et al., 2018).

#### An Hypothesis About Agency (and an Embryo of Self-Awareness)

The cerebral-cerebellar circuits are able to produce a consistent perception of one’s own actions, and the perception of one’s own body parts can be easily tricked; this is reminiscent of the ability of the brain to generate feelings by perceiving and internally depicting emotions. Telling a story about this, i.e., a proper explicit reflexive awareness, is another story, but if a brain were able to generate thought (is it?) there would be no reason to doubt that a similar perception of one’s own mental activity would occur. In facing this question, Sellars (1956) proposed to distinguish “sentience” (sensation, phenomenal consciousness), which any brain would be able to generate, from “sapience” – awareness *of*, awareness *that*, – which requires having the appropriate concepts, learning and inferential capacities typically associated a complex symbolic system (“Awareness is a linguistic affair”).

### An Hypothesis on the Emergence of Thought

Given the complexity and sophistication added by language to mental activity, somebody disputes the concept itself of “preverbal” thought: the term “thought” should be reserved for conscious, aware, linguistically explicit thought; the rest should be considered as mere imaginative activity. In any case, the main feature in both thought and imagination is the capability of shifting focus, either following a thread (logical, mnestic, emotional) or just wandering about, driven by (logical, mnestic, emotional) associations. This suggests that the computational basis for imagination and verbal thought is offered by the mechanisms of selective attention and their dynamics. As mentioned above, such dynamics are made possible by the fact that each cerebral circuit may shift from a non-discriminative (*out of focus*) mode to discriminative (and *in focus*) operation.

The paramount bioethical relevance of being able to detect the presence of a consciousness in a patient has prompted innumerable studies aimed at identifying objective means and procedures to quantify the presence and level of consciousness. Tononi’s group (Tononi et al., 2016) reported a particularly intriguing set of results that convincingly convincingly correlated the “degree” of consciousness (from deep coma to alertness) with the “complexity” of cerebral activity: multipolar EEG (mEEG) responses, generated in remote areas of the cortex by a localized transcranial magnetioc stimulation (TMS) pulse, ranged from null or waning echoes (in deep comatose states) to highly correlated responses (sleep), to relevant and only marginally correlated activities in alert subjects. The increased complexity is paralleled by a decreasing compressibility of mEEG recordings, using standard compression algorithms. The authors interpreted this as a sign that consciousness arises from the concomitant independent processing of information by local circuits combined with strong interactivity among such local circuits.

Given selective attention is an essential feature of consciousness, the continuous switching from discriminative to non-discriminative processing in the many circuits of the brain is expected to add a further level of complexity to the combination of local and interconnected processing and the way they interact at each site in the brain.

### An Hypothesis on the Emergence of Reflexivity

Based on the above considerations, the mode of processing by the neurons appears to be *intrinsically relational*. Specific neural circuits are focussed on detecting such relations in strict reference to one’s own body and emotions, i.e., with the self; this gives rise to an internal picture of reality and what is happening, amounting to an inevitably *subjective and personal* experience. This might be considered as a rudimentary form of consciousness: the perception of being there, feeling emotions and performing actions. All animals seem to possess such an ability, which possibly coincides with what Edelman named “primary consciousness”: the perception of being there, generated by the production of an internal image of reality and its relation with oneself, *here and now* (Edelman and Seth, 2009).

Climbing up the evolutionary scale, with the development of the neocortex such ability becomes ever more detailed, comprehensive and precise. In humans, and possibly some other mammals, much more complex functions come about: (i) the realization of object permanence, i.e., the awareness that reality and objects (and people) remain there and continue to undergo changes even if not perceived, and (ii) the way time is handled by humans, by perceiving it as a line along which things happen and evolve, guided by object permanence, continuity and causality. Object permanence is not present in lower mammals; it is presumably present in primates and possibly some other species; even in humans, it is not an innate capability, but appears to come about during development, by the end of the second year of life, according to Piaget’s (1954) studies, or quite earlier, 9–10 months age, according to more recent studies (Moore and Meltzoff, 2008).

In principle, object permanence and efficient handling of time may be sufficient to transform a “primary consciousness” into the capability of perceiving reality as an evolving system and *telling a story about* reality and oneself, thereby transforming the *awareness of being here, now*, into a diachronic picture of the self as a unitary subject that persists and evolves through time, in a reality that also evolves according to criteria of causality that can somehow be understood: a unitary subject and a reality that have a past, which can be told, and a possible future, which can be imagined, prefigured and possibly determined. Edelman referred to such capability of building a diachronic picture of reality and integrated image of the self as the “higher-order consciousness” typical of humans (Edelman and Seth, 2009).

If “primary consciousness” arises from the capability of producing an internal image of reality, the human brain transforms such image into the perception that reality evolves according to laws of persistence and causal relations, the self is perceived as a diachronic entity with its own story, a present and a future, and the internal picture of reality becomes a story to be told.

Even the most primordial brain is able to interpret each sensory experience as pleasurable, indifferent or noxious, i.e., to generate the raw basis of emotions. In brains with a cortex, the ability to analyze what is there and happens in relational terms, with reference to oneself and one’s own emotions and purposes, emerges from the organization and processing mode of neural circuits. Human brain can add to this a logical, diachronic and causal interpretation, creating a story about it all, in which the protagonist is the subject who sees, interprets, feels, thinks and acts. As Gazzaniga (1998) neatly put it: “the human left hemisphere has the interpreter [whose] job is to interpret our behavior and our responses… It constantly establishes a running narrative of our actions, emotions, thoughts, and dreams.” Such emergent property involves the autoscopic capacity we refer to with the term *reflexivity*.

## Is This “Human Consciousness?”

This exceeds the aim of this discussion. However, the capability of handling symbols and generating a whole symbolic system (language) makes it possible to precisely depict and communicate (apart from the inefficiencies of any communication) one’s own perception of reality. This adds a further dimension to human “higher order consciousness”: in addition to a “primary” perception of the self, here and now, humans perceive their own ability to subjectively tell a story about reality and themselves, *as if one were a spectator of what happens in one’s own brain*. This may possibly be the origin of the autoscopic dimension of consciousness (self-consciousness) and of “Descartes’ error” (Damasio, 1994), the dualistic view of consciousness as a spectator in the theater where the brain sets up its dramas.

Phenomenally conscious states have been defined as states such that “there’s something *it’s like for one* to be in them.” Neurologically, this almost directly translates into neural activity patterns being focussed on by selective attention. Such patterns would be very similar whether they are elicited by sensory inputs or by endogenous activity (imagination), although subcortical neurons and projections to the cortex (mostly serotonergic) normally differentiate the two situations. These internal images (“mental states” or acts) are there whether or not they are targeted by selective attention, although they would be vague and ambiguous like images in mind wandering, day-dreaming, half-awake states (“threshold consciousness”) or dreams, when the cortical structures implied work in a non-discriminative mode. Targeting by selective attention would turn the corresponding circuits into discriminative operation and simultaneously focus and sharpen the internal images, restrict their possible meanings, eliminate ambiguity. This suggests that consciousness consists in some kind of higher order representation – the mind “scanning” itself – that does not require a “higher-order thought,” but a form of representation somehow *sensory* in character, rather than thought-like or conceptual (Lycan, 2004). This can be seen as “a mental state which then becomes the object of a meta-state” (Gennaro, 2005), i.e., a cortical activity pattern which becomes the object of selective attention processes. This idea of mental reflexivity can be tracked back to Kant’s (1787) “inner sense” or Locke’s (1690) “perception of what passes in a man’s mind.” However, quite a number of authors have argued against the idea that this kind of mental reflexivity can account for all that consciousness is: – can account for all that consciousness is (see, e.g., Smith, 2005; Zahavi, 2005).

## Conclusion

Introspection elicits the impression that some kind of duality exists in our consciousness, as if we were, at the same time, the subject of our own life and an external observer who witnesses our life and can tell the story. The way this precisely occurs in the brain remains a challenging, formidable conundrum, still far from solved. On the other hand, “consciousness” is itself an ill-defined term that simultaneously refers to a number of functions – from reactivity to stimuli to alertness and presence, from orientation in time and space to cognitive performance, from correct exam of reality to soundness of thought. Each of these aspects likely involves distinct cerebral circuits and interactions among several cortical areas. Up to now, in the search for a neural correlate of consciousness, which might offer us a way to objectively measure the presence and extent of consciousness (e.g., in vegetative coma or in locked-in syndromes), the most advanced and promising studies point to measures of complexity in EEG recordings; such complexity would arise from the simultaneous operation of many local circuits and computations, with a marked margin of autonomy but strongly interconnected in such a way that partial correlation and partial independence coexist in the activity of the various circuits (Casarotto et al., 2016). Further complexity is added by the mechanisms of selective attention, that produce continuous changes in the mode of elaboration by the various areas of the cortex, sustained by the cortico-thalamic dialogue (Mease et al., 2014), and account for the incessant change of the focus of our consciousness.

Rather than discussing the possible biological substrate of all the cerebral functions that may sustain consciousness, our purpose here was to examine the typical functioning mode of neurons and the way sensory information is processed. This revealed that neuronal processing is intrinsically *relational*, and in particular it tends to be *self-referred*. Furthermore, any activity in the brain tends to be evaluated in terms of vital, emotional and hedonic value, so that neuronal processing inevitably involves the attribution of a meaning (value for one’s survival and well-being) that is essentially *subjective* and *personal*. Cortical areas that have traditionally been considered dedicated to sensory elaboration (such as the parietal cortex) must actually be seen as sensory-motor areas and play a role in motor programming by proposing appropriate *interaction* with any identified object; thus the meaning of any object and situation which is encountered will also be enriched by the judgment of its possible *operative usefulness* (which again adds *subjective* and *personal* relevance). All information collected by the cortex and dissected by the analysis of elements, objects, relations and localisation with respect to the body is conveyed to a structure – the hippocampus – that transforms such streams of data, and the related emotional marks, into an integrated and contextualized *vital experience*.

Hence, we are led to conclude that elaboration by neural circuits necessarily transforms the informational material into a subjective and personal experience. Curiously enough, all this happens independent of any reference to consciousness, except for the most raw and elementary aspect of consciousness, i.e., a sufficient arousal and quantitative level of cortical activity.

Thus, perhaps unexpectedly, subjectivity turns out to be there before – not as a consequence of – consciousness.

If we wish to consider subjectivity as a specific feature of consciousness, we must conclude that it is provided *to* consciousness by the intrinsic operating mode of the brain and not added to the processes going on in the brain by some mysterious biological or metaphysical process or entity (consciousness). Consciousness appears to make humans able to extract from experience what has happened outside and within themselves in the past, what might have happened if only…, what is occurring now and will or may occur in the future; to tell a story about all this; to understand, simulate, imagine and predict. But, contrary to a possible layman’s perception, consciousness – in its somewhat mysterious nature – is not the origin of these abilities or the *source* of an individual perspective on the external reality and the self: because consciousness works on “material” (neurally processed information) that already has in itself a well-defined subjective, emotional and individual characterisation.

## Data Availability Statement

The original contributions presented in the study are included in the article/supplementary material, further inquiries can be directed to the corresponding author.

## Author Contributions

The author confirms being the sole contributor of this work and has approved it for publication.

## Conflict of Interest

The author declares that the research was conducted in the absence of any commercial or financial relationships that could be construed as a potential conflict of interest.
